# Deciphering age-related transcriptomic changes in the mouse retinal pigment epithelium

**DOI:** 10.18632/aging.206219

**Published:** 2025-03-04

**Authors:** Sushil K. Dubey, Rashmi Dubey, Kyungsik Jung, Alvaro G. Hernandez, Mark E. Kleinman

**Affiliations:** 1 Department of Surgery, East Tennessee State University, Johnson City, TN 37614, USA; 2 Roy J. Carver Biotechnology Center, University of Illinois at Urbana-Champaign, Urbana, IL 61801, USA

**Keywords:** transcriptome, retinal pigment epithelium, oxidative stress, inflammation, chronological aging

## Abstract

Aging of the retinal pigment epithelium (RPE) leads to a gradual decline in RPE homeostasis over time, significantly impacting retinal health. Understanding the mechanisms underlying RPE aging is crucial for elucidating the background in which many age-related retinal pathologies develop. In this study, we compared the transcriptomes of young and aged mouse RPE and observed a marked upregulation of immunogenic, proinflammatory, and oxidative stress genes in aging RPE. Additionally, aging RPE exhibited dysregulation of pathways associated with visual perception and extracellular matrix production. Research on aging in post-natal quiescent RPE is hindered by the absence of relevant *in vitro* models. Here, we evaluated an *in vitro* model of chronologically aged primary human RPE to address this gap and observed gene expression patterns comparable to native-aged RPE. Gene expression profiling in this model highlighted its potential utility in investigating cellular and molecular mechanisms of RPE aging and in screening of therapeutic compounds. In conclusion, our findings underscore the pivotal role of inflammation, immune activation, and oxidative stress in the aging RPE landscape and provide insights into why age increases the risk of retinal pathologies.

## INTRODUCTION

The retinal pigment epithelium (RPE) is a monolayer of highly polarized cells positioned between the neuroretinal photoreceptors and the choroidal capillaries [1]. Originating from neuroectoderm, these post-mitotic cells display highly convoluted basal infoldings that attach to a specialized Bruch’s basement membrane which is an acellular layer separating the RPE from the choriocapillaris. The RPE forms a critical physiologic barrier in the outer retina offering multifunctional support to the photoreceptor layer and facilitating essential visual cycle metabolism [1, 2]. The RPE plays a vital role in retinal homeostasis by maintaining the integrity of the outer-retina blood barrier and facilitating nutrient and oxygen diffusion from blood to photoreceptors. A core function of the RPE entails the processing of visual cycle intermediates with the continuous exchange of retinal chromophores between photoreceptors and the RPE during which the RPE re-isomerizes all-trans-retinal to 11-cis-retinal and transports it back to photoreceptors [3, 4]. This process is central to the visual cycle and sustains photoreceptor function. Furthermore, the RPE stabilizes ion composition in the subretinal space through voltage-dependent ion conductance in its apical membrane which is also essential for maintaining photoreceptor excitability and viability [5]. Another critical role of the RPE is the phagocytosis of shed photoreceptor outer segments wherein the outer segments are digested and essential substances such as retinaldehyde are recycled and returned to photoreceptors to replenish light-sensitive outer segments [6]. Therefore, any damage and dysfunction in the RPE deleteriously affect the health of photoreceptors, degrade retinal function, and ultimately lead to vision loss.

During the physiological aging process, retinal tissues undergo functional decline and degeneration with the RPE serving as the primary site of damage in many age-related retinal diseases [7]. While aging itself may not invariably lead to the onset of conditions such as age-related macular degeneration (AMD), retinitis pigmentosa (RP), and diabetic retinopathy (DR), age-related changes can predispose the eye to these diseases [8]. AMD, a leading cause of vision loss in developed countries, is characterized by progressive degeneration of the RPE, retina, and choriocapillaris [9, 10]. Numerous studies have documented age-related physiological changes in the RPE and the underlying Bruch’s membrane, including the accumulation of intracellular granules containing oxidized lipids known as lipofuscin, mitochondrial DNA damage, lysosomal dysfunction, buildup of metabolic debris, and drusen biogenesis which is a hallmark feature of AMD progression [11–14]. Drusen accumulation impedes diffusion from the choroidal circulation to the retina exerting adverse effects on both RPE and photoreceptors. Drusen has a complex composition that comprises many inflammatory proteins, including complement factors, cytokines, C-reactive protein, IgG, and major histocompatibility class II molecules [15, 16]. Elevated expression and localization of proinflammatory factors associated with drusen biogenesis trigger oxidative stress and cellular dysfunction, which have long been recognized as significant factors influencing the pathophysiology of AMD [12, 17]. Due to the neural retina’s high energy requirements, the RPE remains metabolically active, constantly producing energy to support both the neural retina and the visual cycle. As a result, the RPE is abundant in mitochondria, making it a primary source of reactive oxygen species (ROS), and these mitochondria increase their ROS production during aging [18]. Despite scientific advancements that have established a multitude of molecular pathways significant in the pathogenesis of RPE atrophy and age-related retinal diseases, the genes driving biologic aging remain poorly understood [19, 20]. Deciphering differential gene expression profiles in young and aged RPE is critical to identifying molecular mechanisms that provide fundamental scientific insight into RPE aging and potential therapeutic targets for age-related retinal pathologies.

Chronological aging models of laboratory animals provide an extremely powerful experimental approach to studying molecular mechanisms that contribute to the development of age-related retinal diseases. Utilizing transcriptome-wide RNA sequencing, we studied differential gene expression profiles in the RPE/choroid of young and aged mice and identified global transcriptomic changes underlying the biologic aging of the RPE. The molecular pathways significantly enhanced in aged RPE suggest a predisposition in older mice towards inflammation, immune activation, and oxidative stress, which are known drivers of age-related retinal pathologies. In a correlated *in vitro* model of primary human RPE isolates, chronological aging similarly resulted in gene expression profiles associated with increased inflammatory and oxidative stress responses. These findings significantly expand our fundamental scientific understanding of age-related retinal diseases while offering potential new biomarkers for clinical diagnosis and therapeutic strategies to reverse biologic aging in the RPE.

## RESULTS

### Transcriptome profiling of young and aged mouse RPE/choroid

**Figure 1:**
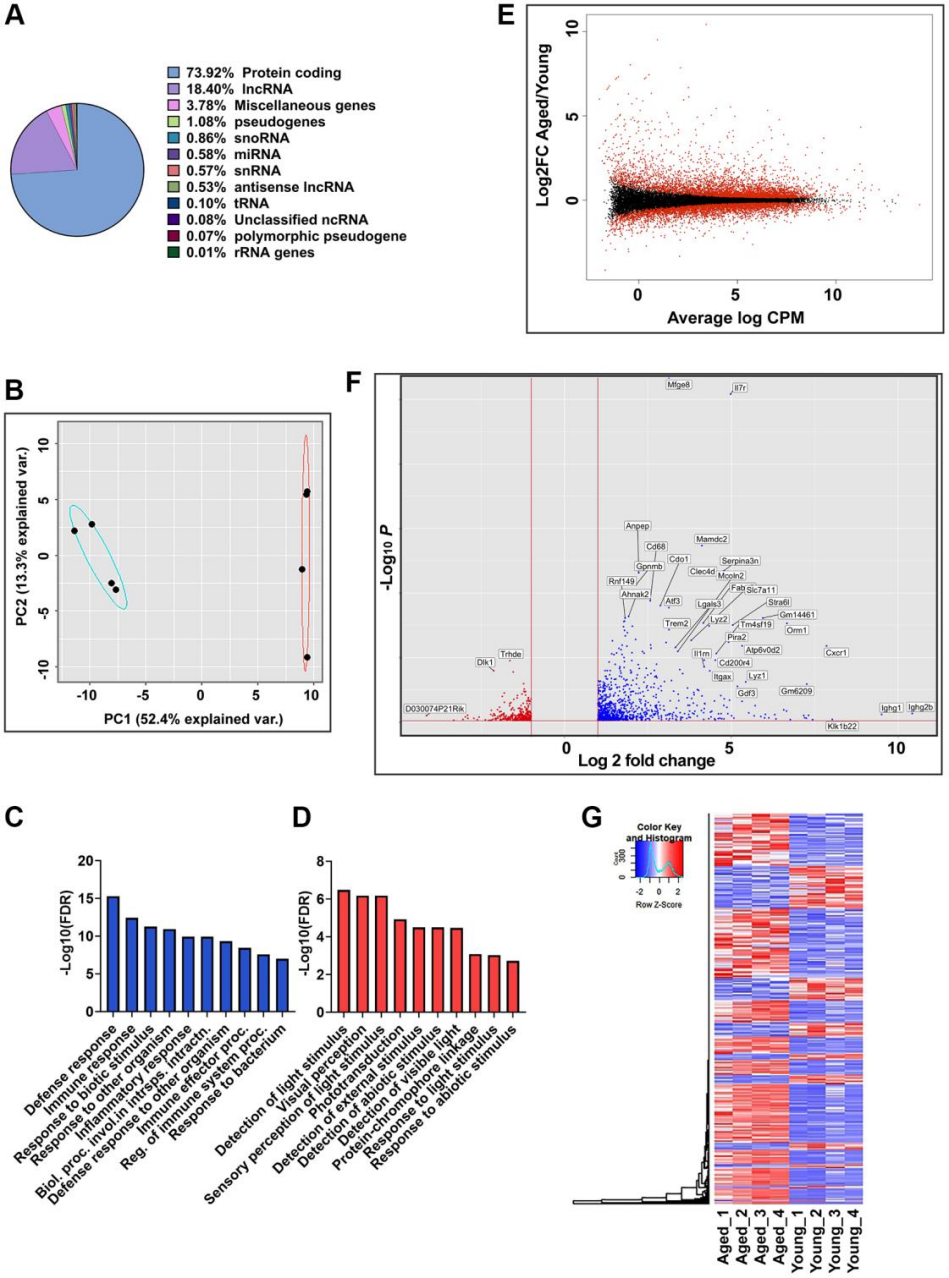
**Global gene expression analysis in RPE/choroid of young and aged mice.** (**A**) Pie chart represents the percent of globally expressed transcript subspecies across young (2–3 months, *n* = 4) and aged (22–24 months, *n* = 4) mice RPE/choroid from 21,376 annotated transcripts. (**B**) PCA plots of whole transcriptome data showed distinct clustering of young and aged mice along PC1, which captures the maximum variance (52.4%). Blue and red ellipses indicate young and aged mice and black dots represent the biological replicates (*n* = 4). (**C**, **D**) GO enrichment terms associated with the top 100 PC1 positive loading genes (**C**) and PC1 negative loading genes (**D**). (**E**) MA plot of log2 fold change versus average log counts-per-million (CPM) based on edgeR analysis showing the differential gene expression between aged and young mice RPE/choroid. Red dots indicate differentially expressed genes (FDR ≤0.05), and black dots indicate non-differentially expressed genes. (**F**) Volcano plot showing genes significantly (*p*-adj value < 0.05) upregulated (red) and downregulated (blue) in aged mice RPE/choroid. The x-axis represents log2-fold change, and the y-axis represents −log10 (*p*-value). The dotted line shows a cutoff of −log10 (*p*-value) < 0.05. Annotated dots represent the top significantly regulated genes. (**G**) Hierarchical clustering and heatmap analysis of gene expression in the RPE/choroid tissues of the young vs. aged mice. Blue to red represents low to high gene expression.

RNA-seq data were generated from RPE/choroid tissues of both young (2–3 months) and aged (20–24 months) C57BL/6J mice (*n* = 4, two males and two females per group). This comprehensive approach aimed to capture the entire transcriptome profile and provide insights into age-related changes in gene expression in RPE/choroid. The sequencing depth averaged 50.2 million reads per library, with 21,376 transcripts mapped to the mouse reference genome (GRCm39). Of the 21,376 transcripts mapped, 73.92% were protein-coding, and 18.4% were assigned to long non-coding transcript types, making them the predominant transcript types (Figure 1A). The remaining ~3.8% represent other transcript types such as snoRNAs, miRNAs, snRNAs, unclassified ncRNAs, antisense lncRNAs, tRNA, rRNA, pseudogenes, and polymorphic pseudogenes (Figure 1A). Principal component analysis (PCA) was performed using the normalized read counts to obtain a global characterization of the mouse transcriptome. PCA revealed distinct clustering of young and aged groups along PC1, accounting for 52.4% of the variance, suggesting age as the primary driving factor (Figure 1B). To elucidate the biological processes underlying this segregation, Gene Ontology (GO) analysis was performed on the top 100 loading genes of PC1. Genes with positive loadings in PC1 exhibited enrichment in GO terms related to defense response, immune response, and inflammatory response pathways (Figure 1C), while genes with negative loadings in PC1 were enriched for GO terms associated with visual perception and phototransduction (Figure 1D and Supplementary Table 1).

Differential expression analysis using edgeR identified significant alterations in 5,469 genes between young and aged RPE/choroid samples (adjusted *p*-value < 0.05). The gene expression distribution across the entire transcriptome dataset was depicted through an MA plot (Figure 1E). Thresholds of the adjusted *p*-value < 0.05 and log2 fold change (Log_2_FC) ≤−1 and ≥+1 were applied to define differentially expressed genes (DEGs). Analysis revealed 1,300 DEGs, including 939 upregulated and 361 downregulated genes in aged RPE compared to young RPE. A volcano plot illustrated the magnitude of differential expression (Figure 1F) with higher transcriptional activation in aging mouse RPE/choroid, as measured by both the number of DEGs induced and their Log_2_FCs. Furthermore, hierarchical clustering and heatmap analyses of these DEGs illustrated distinct expression profiles between young and aged groups consistent with age-associated alterations in RPE gene expression (Figure 1G).

### Gene enrichment analysis reveals induction of immune and inflammatory response in aged mouse RPE/choroid

**Figure 2:**
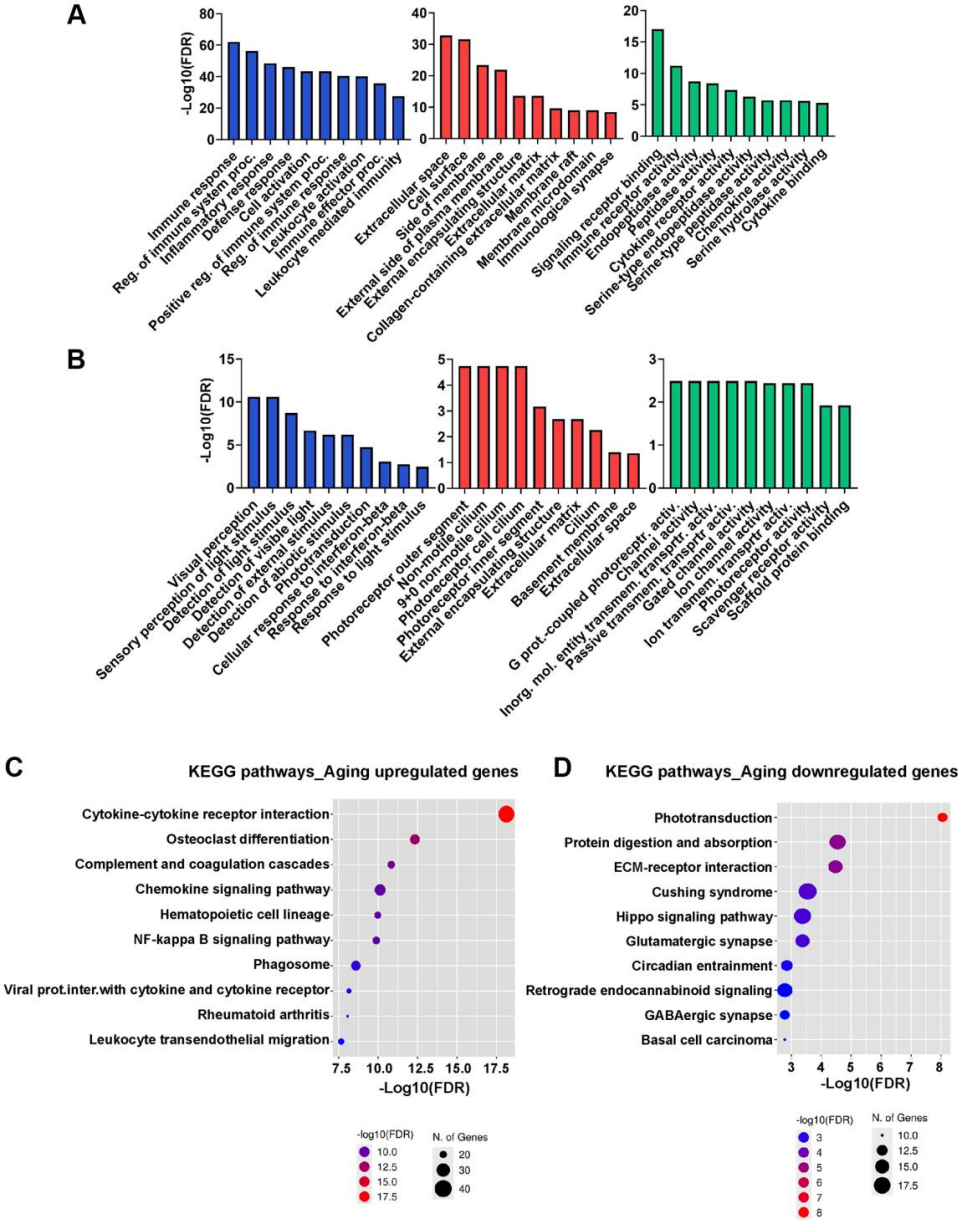
**GO and KEGG functional enrichment analysis.** (**A**, **B**) GO analysis of upregulated (**A**) and downregulated (**B**) genes in aged RPE/choroid in the BP, CC, and MF categories. The x-axis displays the top 10 most significant GO terms, and the y-axis represents the -log10 (FDR) of the enriched terms. (**C**, **D**) Dot plot showing KEGG pathway enrichment analysis of upregulated (**C**) and downregulated (**D**) genes in aged RPE/choroid. The y-axis presents the names of the top 10 enriched pathways, and the x-axis represents the -log 10 (FDR). The number of DEGs enriched in a pathway is denoted by bubble size, and the -log10 (FDR) is reflected by the bubble’s color. Abbreviations: DEGs: differentially expressed genes; GO: Gene Ontology; CC: cellular component; MF: molecular function; BP: biological process; KEGG: Kyoto Encyclopedia of Genes and Genomes.

To further elucidate the characteristics of the DEGs, we performed a GO analysis using the top 100 significantly upregulated and downregulated genes. The functional GO terms were classified into three categories: biological process (BP), cellular component (CC), and molecular function (MF), as shown in Figure 2A, 2B. For genes upregulated in aged RPE, GO analysis revealed a notable enrichment in biological processes associated with immune response, regulation of immune system processes, inflammatory response, defense response, cell activation, positive regulation of immune system processes, regulation of immune response, leukocyte activation, immune effector processes, and leukocyte-mediated immunity. Additionally, molecular function and cellular component GO terms displayed enrichment in signaling, receptor activity, extracellular space, and cell surface, indicative of active proinflammatory milieu in the aging RPE (Figure 2A and Supplementary Table 2). GO annotation of downregulated genes included processes related to visual perception, sensory perception of light stimulus, detection of light stimulus, detection of visible light, detection of external stimulus, detection of abiotic stimulus, phototransduction, cellular response to interferon-beta, response to interferon-beta, and response to light stimulus (Figure 2B). This decline in the expression of genes associated with visual pathways and phototransduction is consistent with the potential degeneration of RPE and impaired vision associated with aging. Photoreceptor outer segment and G-protein coupled photoreceptor activity were the most significantly enriched GO terms in the cellular component and molecular function categories, respectively (Figure 2B and Supplementary Table 2). These findings suggest that genes implicated in crucial RPE functions, such as maintaining visual processing in the retina including absorption of light passing through the neurosensory retina, phagocytosis of photoreceptor outer segments, and sustaining the retinoid cycle or visual cycle are impacted by aging.

DEGs were then mapped to their biologically relevant pathways using the KEGG pathway database. Among the ten most significantly upregulated pathways in aging RPE, the majority were immune-related (Figure 2C) with the cytokine-cytokine receptor pathway being the most enriched (*p*-value: 7.4E-19; Supplementary Figure 1). This finding aligns well with the results of the GO analysis, as cytokine production is known to be highly responsive to inflammation in the RPE [21]. Within this pathway, a total of 94 genes exhibited significant alterations in aged RPE/choroid comprising both receptor and ligand molecules including numerous chemokines, cytokines, interleukins, TNF, and TGF factors. This extensive modification underscores the presence of a robust autocrine and paracrine signaling milieu in aging RPE (Supplementary Figure 1). Other prominent age-related networks that emerged from KEGG analysis were osteoclast differentiation, complement activation, and coagulation cascades (Figure 2C). In contrast, downregulated genes did not exhibit significant enrichment in any pathways, potentially due to many of these genes lacking annotations in specific KEGG pathways. Therefore, we expanded our analysis to include 1,418 genes using a threshold of adjusted *p*-value < 0.05 and Log_2_FC ≥0.5 for KEGG analysis. Among the top three downregulated pathways were phototransduction, protein digestion and absorption, and extracellular matrix (ECM)-receptor interaction (Figure 2D). The downregulation of the phototransduction pathway and dysregulation of protein turnover emerged as key processes in aging RPE. Furthermore, the loss of ECM-receptor interaction underscores a hallmark of RPE aging which likely contributes to the disorganization of the ECM [22].

### Establishment of PPI networks and hub gene analysis

**Figure 3:**
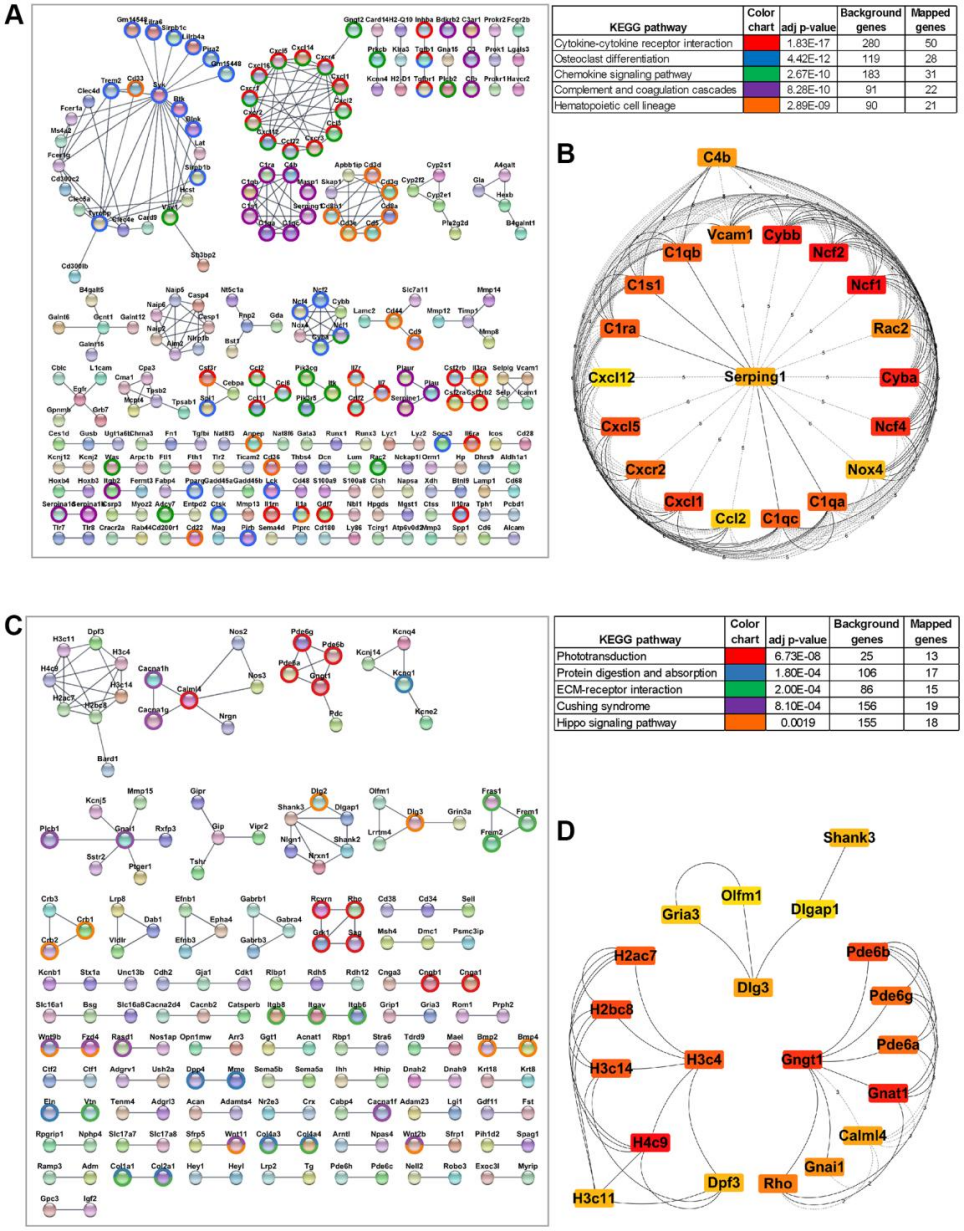
**Protein-protein interaction (PPI) network of differentially expressed genes and the hub genes.** (**A**) STRING network of PPI generated using DE genes upregulated in aging RPE/choroid (FDR <0.05, log2 fold change >1). Significant clusters from the PPI network complex were constructed using the Cytoscape MCL plugin. Genes mapped to top KEGG pathways are highlighted. (**B**) The top 20 hub genes in the PPI network were identified using the Cytoscape plugin cytoHubba based on their maximal clique centrality (MCC) score. The 20 identified hub genes are displayed from red (high MCC score) to yellow (low MCC score). (**C**) PPI network constructed using DE genes downregulated in aging RPE/choroid (FDR <0.05, log2-fold change >0.05). The network is subclustered using the Cytoscape MCL plugin and the highlighted genes are mapped to top KEGG pathways. (**D**) Top 20 aging downregulated hub genes screened by the cytoHubba plugin of Cytoscape based on the MCC score where red nodes represent a higher MCC score and yellow represents a lower score.

We constructed a Protein-Protein Interaction (PPI) network utilizing the STRING database (v12.0) and Cytoscape software to evaluate the relationship among the upregulated DEGs in the aging RPE [23]. To ensure the reliability of the PPIs, we set a stringent cutoff threshold of a high confidence interaction score of ≥0.9, resulting in a robust PPI network comprising 726 nodes and 437 edges. Using the Cytoscape MCL mode, DEGs were subclustered within the PPI network, with the top five significant KEGG pathways assigned to distinct clusters (Figure 3A). Subsequently, interactions within the network were leveraged to identify hub genes using the Maximal Clique Centrality (MCC) algorithm in the CytoHubba plugin [24] of Cytoscape. In this analysis, genes with the top 20 MCC scores were designated as hub genes (Figure 3B). Among these 20 hub genes were key players involved in the NADPH oxidase complex (*Cyba, Cybb, Ncf1, Ncf2, Ncf4, Nox4, Rac2*), the complement pathway (*C1qa, C1qb, C1qc, C1ra, C1s1, C4b, Serping1*), and chemokines and chemokine receptors (*Ccl2, Cxcl1, Cxcl12, Cxcl5, Cxcr2*) (Figure 3B).

A similar analysis was performed using the 1,418 DE genes (*p*-value < 0.05 and Log_2_FC ≥0.5) downregulated in aging RPE. These genes were integrated into a coexpression network at a cutoff threshold of ≥0.9 confidence interaction score utilizing STRING and Cytoscape with 776 nodes and 181 edges. The DEGs were further subclustered using the Cytoscape MCL function and mapped to various KEGG pathways (Figure 3C). The most significant cluster, comprising 20 hub genes, was identified using the MCC algorithm (CytoHubba), consisting of genes central to chromatin organization (*H2ac7, H2bc8, H3c11, H3c14, H3c4, H4c9, Dpf3*), visual perception (*Pde6a, Pde6b, Pde6g, Gnat1, Calml4, Gnai1, Rho and Gngt1*), and neuronal synapse genes (*Shank3, Dlg3, Dlgap1, Olfm1, Gria3*) (Figure 3D).

### Validation of RNA-seq profiles by qPCR

**Figure 4:**
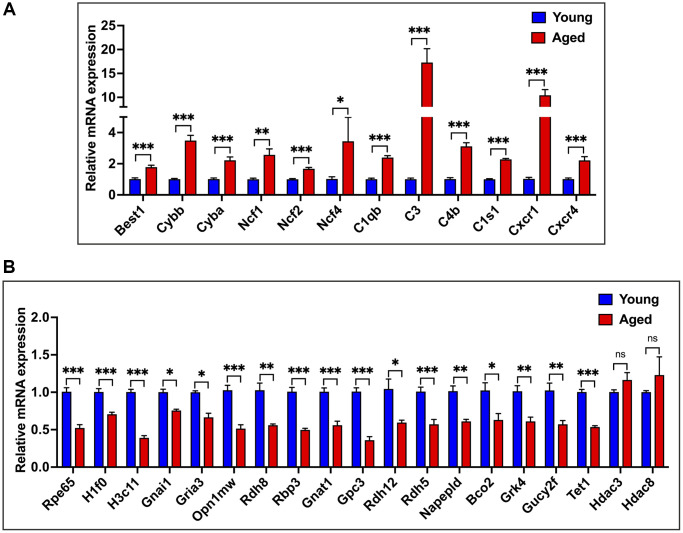
**qPCR validation of RNA-seq data.** (**A**) qPCR expression analysis of 12 genes upregulated in aged RPE/choroid. (**B**) Relative mRNA expression levels of genes downregulated in aging and not differentially regulated in RPE/choroid from young and aged mice. Actin-B was used for normalization, and statistical analysis was performed using the unpaired *t*-test (^*^p < 0.05, ^**^p < 0.01, ^***^p < 0.001, ns: nonsignificant). Data were presented as mean ± SEM.

We validated our RNA-seq data findings through qPCR analysis using a new cohort of animals. We selected 31 genes: 12 age-upregulated, 17 age-downregulated, and two genes that showed no significant difference between young and aged RPE. The age-upregulated representative genes of major pathways included *Cybb, Cyba, Ncf1, Ncf2,* and *Ncf4* for the oxidative stress and redox pathway, *C1qb, C3*, *C4b, and C1s1* for the complement pathway, *Cxcr1* and *Cxcr4* for chemokine response and *Best1,* an RPE-specific marker (Figure 4A). For transcripts downregulated in aging, we analyzed *Rpe65*, the classical RPE biomarker; histones *H1f0* and *H3c11*; hub genes *Gnai1, Gria3, Gnat1*; visual and phototransduction pathway genes *Opn1mw, Rdh8, Rbp3, Gpc3, Rdh12, Rdh5, Napepld, Bco2, Grk4, Gucy2f,* and the epigenetic regulator *Tet1* (Figure 4B). Additionally, we verified the expression of other epigenetic regulators, *Hdac3* and *Hdac8*, which remained unchanged with age (Figure 4B). The results of these expression analyses corroborated the RNA-seq data. In line with the RNA-seq and qPCR results, immunofluorescence (IF) staining of retinal cross sections in mice showed increased expression of C4b and reduced expression of H1f0 in aged mice RPE compared to young cohorts (Supplementary Figure 2).

### Hub genes mapping and pathway visualization

**Figure 5:**
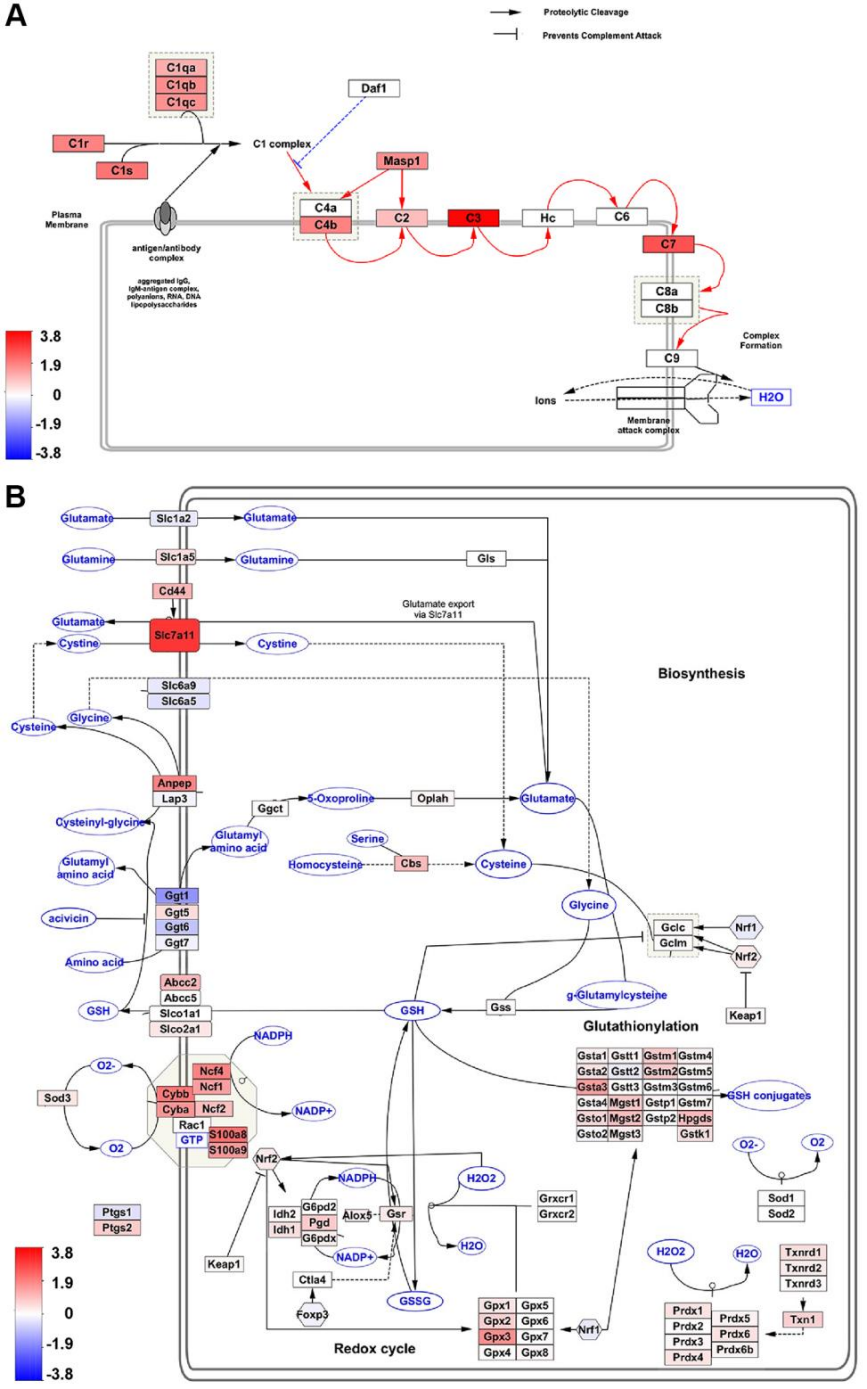
**Pathway analysis of hub genes.** (**A**, **B**) Wikipathway analysis shows Hub gene-associated pathways. The number of DEGs in our dataset that overlap with the complement activation classical pathway (**A**), and oxidative stress and redox pathway (**B**) are indicated as red (aged upregulated) or blue (aged downregulated).

We utilized the WikiPathways app within the Cytoscape network analysis and visualization software to conduct pathway mapping of hub genes. Wikipathways, a curated collection of mouse pathways, was used to identify the biological pathways most affected in RPE with age [25]. The top most significant Wikipathways to which hub genes were mapped included the complement activation classical pathway (WP200, adj *p*-val = 8.3E-10), oxidative stress and redox pathway (WP4466, adj *p*-val = 8.5E-08), and chemokine signaling pathway (WP2292, adj *p*-val = 8.3E-10) (Figure 5A, 5B and Supplementary Figure 1). Moreover, several genes associated with these pivotal pathways were also significantly altered in our transcriptome dataset. The majority of these genes exhibited positive regulation, indicating the crucial involvement of oxidative stress, complement, and the chemokine system in RPE aging (Figure 5A, 5B and Supplementary Figure 1).

### Age-related expression profiles of RPE signature genes

**Figure 6:**
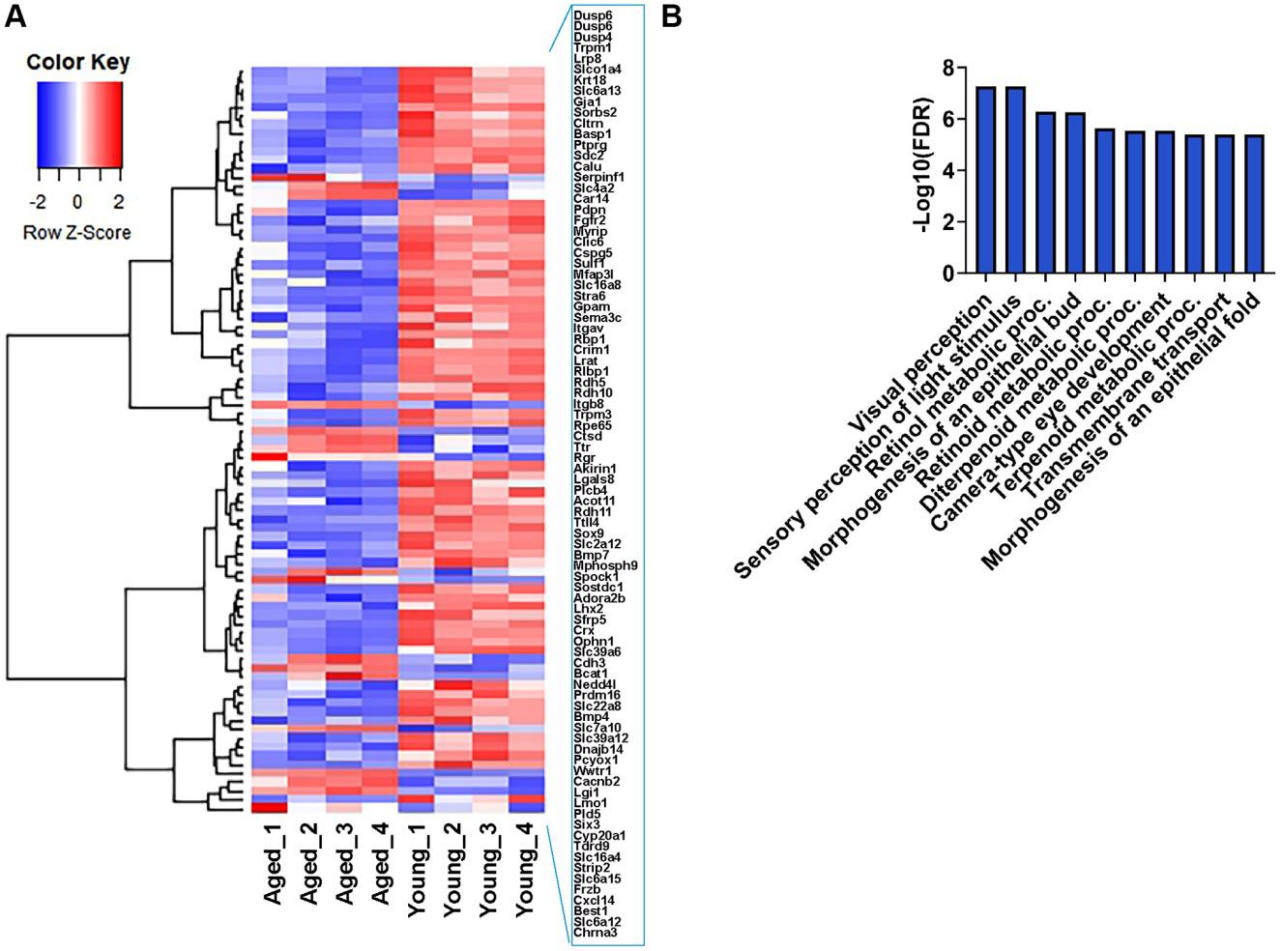
**Expression profile of RPE-specific genes.** (**A**) Hierarchical clustering and heatmap analysis of selected RPE-specific markers in the RPE/choroid of young and aged mice. Blue to red represents low to high gene expression. (**B**) GO term enrichment analysis of 67 RPE-specific genes that were downregulated in aging.

A comprehensive study by Bergen et al. compiled a list of 171 human RPE-specific markers [26]. We performed a comparative analysis between these RPE signature genes and our transcriptome data to identify age-related changes. Of these, 162 genes aligned with our dataset, revealing significant differential gene expression in 85 RPE marker genes between young and aged mice. The heatmap showed that a significant number of these genes were downregulated in aging RPE (Figure 6A). GO analysis indicated that 67 downregulated genes were involved in visual perception, sensory perception of light stimulus, retinol metabolic process, morphogenesis of an epithelial bud, retinoid metabolic process, diterpenoid metabolic process, camera-type eye development, terpenoid metabolic process, transmembrane transport, and morphogenesis of an epithelial fold, consistent with declining functional ability and metabolic processes in the aged eye (Figure 6B). Among these 171 RPE-specific signature genes, 77 exhibited no significant changes in expression, and these included genes enriched in cytoskeleton organization and spindle organization (data not shown). Although these genes remained unchanged between young and aged RPE, they highlight the biological processes that remain unaltered with advancing age. Therefore, these genes are probably more robust RPE-specific markers in the context of aging and senescence.

### Acquisition of inflammatory and immune active phenotypes by *in vitro* hRPE aging models

**Figure 7:**
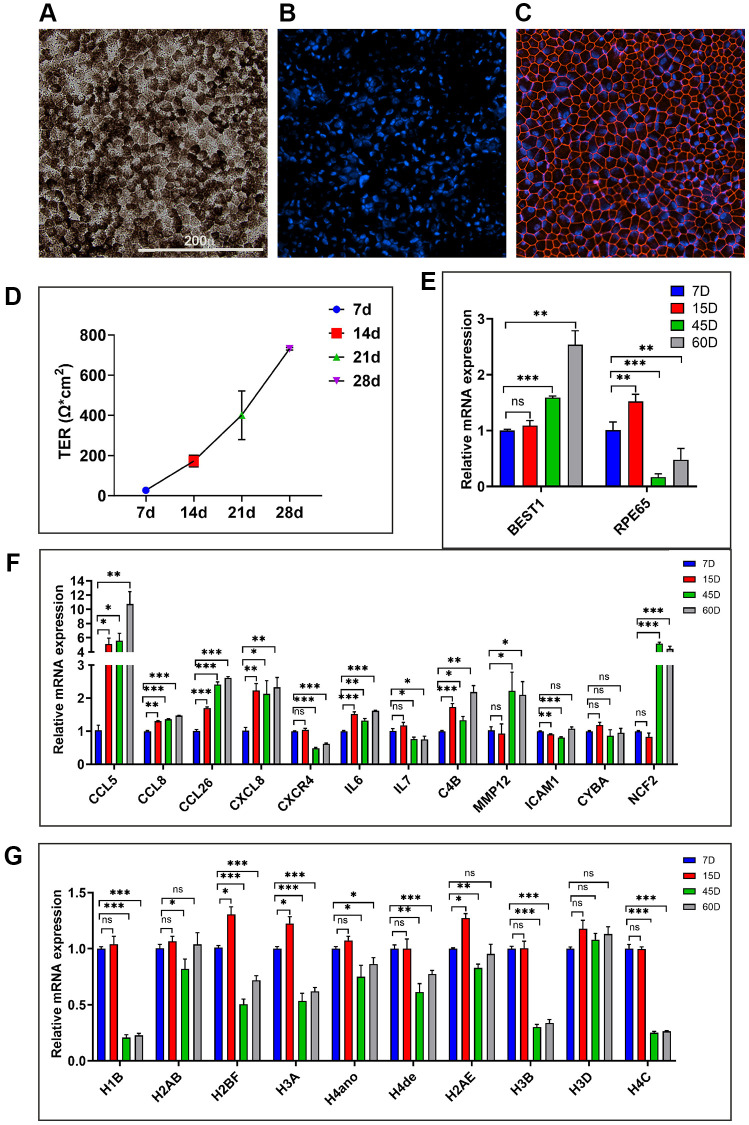
**Evaluating the *in vitro* hRPE aging model.** (**A**) Representative image of 21-day hRPE confluent cultures exhibit pigmentation and hexagonal cellular monolayers. (**B**, **C**) Representative immunofluorescence staining for the tight junction marker ZO-1 (Red) on day 21 and the nuclei are co-stained with Hoechst (Blue). (**D**) Time-dependent increase in TER from multiple transwells containing long-term primary hRPE cultures measured at 7/14/21 and 28 days. (**E**) Expression of RPE marker genes, *BEST1* and *RPE65* in the long-term hRPE cultures. (**F**, **G**) Relative mRNA expression levels of genes are significantly upregulated and downregulated in long-term hRPE cultures. Actin-B was used for normalization, and statistical analysis was performed using the unpaired *t*-test (^*^p < 0.05, ^**^p < 0.01, ^***^p < 0.001, ns: nonsignificant). Data were presented as mean ± SEM.

Next, we utilized long-term primary human RPE (hRPE) cultures (*n* = 3 isolates) to evaluate their suitability as a model for studying RPE aging by comparing their characteristics with *in vivo* models. To establish *in vitro* aged RPE cultures from human donors, isolated RPE cells were cultured for up to 60 days according to the protocol outlined in the methods section. By day 2, these long-term cultures exhibited 100% confluency and a hexagonal lattice morphology reminiscent of native RPE tissue *in vivo*. Long-term culture of hRPE developed dense pigmentation after approximately 21 days (Figure 7A). Confocal immunofluorescent images of 21-day cultures stained positively for the tight junction protein, ZO-1, indicating continuous junctions between cells (Figure 7B, 7C). Weekly measurements of transepithelial resistance (TER) revealed a progressive increase, with hRPE cells (*n* = 3) developing a maximum resistance of 731.2 (±6.6) Ω.cm^2^ at 28 days. Resistances ranging from 172.3 (±28.8) to 731.2 (±6.6) Ω.cm^2^, observed between days 14 and 21 in this long-term culture, surpassed the typical TER values of the human RPE monolayer (150 Ω.cm^2^) *in vivo* [27] (Figure 7D). This suggests that the hRPE cells achieved functional polarization and tight junction formation. While the long-term cultures expressed RPE markers RPE65 and BEST1, the expression of these markers was altered with increasing culture time (Figure 7E).

As many previous studies [28, 29] have identified the 21-day timepoint as optimal for establishing long-term RPE cultures, we extended culturing hRPE cells to 45 and 60 days to obtain aged cells. To evaluate whether the long-term primary hRPE cultures can serve as a model for RPE aging, we used qPCR to assess a panel of proinflammatory, immune response, matrix remodeling, and oxidative stress-related genes from cultures of 7/15/45 and 60 days (Figure 7F). These genes were significantly altered with age in our *in vivo* transcriptomic data. Furthermore, a subset of these genes was also found to be significantly altered in the *in vitro* replicative model of hRPE aging, as reported in our recent study [30]. We examined the time-course expression trend of *CCL5, CCL8, CCL26, CXCL8, CXCR4, IL6, C4B, MMP12, ICAM1, CYBA*, and *NCF2* genes. In accordance with our *in vivo* aging RPE data, *CCL5, CCL8, CCL26, CXCL8, IL6, C4B, MMP12,* and *NCF2* genes exhibited a progressive increase in expression across the time course analysis (Figure 7F). These findings indicate the acquisition of an inflammatory and immunologically active phenotype, along with upregulation of redox stress response in chronologically aged *in vitro* hRPE cultures. While *CXCR4* showed a significant downregulation across different time points, *ICAM1* and *CYBA* showed no change in expression levels.

We analyzed histone expression levels in chronologically aged hRPE cells, building upon our recent demonstration of significant histone downregulation in aging mouse and human RPE *in vivo* [30]. A progressive depletion of various histone isoforms was observed at 15, 45, and 60 days (Figure 7G) compared to 7-day controls. Certain histone isoforms like *H1B, H2BF, H3A, H3B,* and *H4C* were substantially depleted with increasing culture time, unlike *H2AB* and *H3D,* which remain unchanged. Together, these findings highlight that chronological aging of RPE, both *in vivo* and *in vitro*, is marked by the upregulation of inflammation genes and oxidative stress genes. Our model of long-term hRPE cultures recapitulated many validated cellular and molecular signatures of the aging RPE *in vivo*.

## DISCUSSION

The primary objective of the current study was to elucidate the gene expression changes occurring during the natural aging process of the RPE by comparing transcriptome profiles between young and aged mice. Given the central role of RPE in age-related pathologies like AMD, understanding the precise molecular changes that take place in the RPE during aging is imperative [31]. Our analysis revealed divergence in the transcriptomes of RPE tissues from young and aged mice, as evidenced by the PCA. Notably, chronological aging emerged as the primary source of variation in the entire dataset, predominantly attributed to PC1. Functional enrichment of the genes driving PC1 identified immune and inflammation-related genes as the principal drivers of age-related alterations in gene expression. This observation aligns with previous research indicating the emergence of a low-grade inflammatory state known as the senescence-associated secretory phenotype (SASP) in aging mouse RPE [30].

In our study, a substantial number of protein-coding transcripts exhibited significant upregulation in the RPE of aging mice compared to their younger counterparts. This heightened transcriptional activity was reflected in the upregulation of numerous genes encoding immune regulators and proinflammatory factors across cellular locations, including the extracellular space, cell surface, membrane, and extracellular matrix. Similar patterns of increased immune and inflammatory responses have been noted in studies examining aging RPE in humans and non-human primates [7, 32]. Polarized RPE cells interact with the ECM-rich interphotoreceptor matrix and outer segments of photoreceptor cells at the apical surface and with Bruch’s membrane at the basal surface. While the normal RPE actively participates in the ECM synthesis, with advancing age, the ECM microenvironment undergoes substantial changes compromising RPE homeostasis and function [33]. This alteration leads to diminished cell adhesion, proliferation, and migration, as well as reduced phagocytosis of photoreceptor outer segments. Moreover, the evolving ECM landscape influences ECM-associated immune and inflammatory processes [34, 35]. Lastly, the identification of abundant long non-coding RNAs (lncRNAs) reveals another layer of complexity governing the transcriptomic milieu of RPE cells and warrants exploration in future investigations.

GO enrichment analysis of DE genes downregulated in aging revealed biological processes related to the regulation of visual processes, particularly at the RPE-photoreceptor interface. This emphasizes aging as a significant risk factor disrupting the homeostasis of the RPE-photoreceptor system. The RPE plays a crucial role in the visual cycle. Light absorption by rhodopsin in photoreceptors initiates phototransduction, converting 11-cis-retinal to all-trans-retinal (atRAL). This atRAL is then transported to RPE cells where it undergoes conversion back to 11-cis-retinal before being returned to photoreceptors to regenerate rhodopsin, thus completing the cycle [36]. Dysfunctions in the visual cycle result in the accumulation of atRAL, leading to the degeneration of both photoreceptor and RPE cells due to their intimate symbiotic relationship.

Over-representation analysis of DE genes identified functional pathways enriched in both young and aged mice RPE/choroid. The most enriched pathway observed in aged mice RPE/choroid was the cytokine-cytokine receptor pathway. Pathway mapping showed the upregulation of diverse groups of chemotactic cytokines and chemokines that belong to CC, CXC, C families and their receptors. Several members of the interleukins (IL), interferons (IFN), tumor necrosis factor (TNF), and Transforming Growth Factor (TGF) family upregulated in aging RPE were also mapped to the pathway (Supplementary Figure 1). Members of the cytokine family have been implicated in the recruitment of immune cells and activation of inflammatory pathways that ultimately contribute to RPE degeneration and AMD pathogenesis. Other pathways, such as osteoclast differentiation, complement activation, coagulation cascades, chemokine signaling, and hematopoietic cell lineage were also upregulated in aged RPE/choroid. Previous studies have reported some common genes involved in RPE reprogramming and osteoclast differentiation [37, 38]. The young RPE/choroid was most enriched in pathways involved in phototransduction and protein digestion and absorption.

The interaction network of the DE genes was used to derive the hub genes, which may be central to RPE aging pathways and could serve as potential biomarkers for the diagnosis and treatment of age-related retinal diseases. In our study, the 20 hub genes include the five subunits of the NADPH oxidase complex, which is directly involved in ROS production [39]. Physiologic ROS production in the RPE is involved in signal transduction cascades, and typically RPE oxidative stress is reduced by endogenous RPE antioxidants and antioxidant enzymes such as superoxide dismutase (SOD) and catalase and glutathione peroxidase (GPX) [40, 41]. With aging, these RPE defenses diminish, allowing heightened ROS production and apoptotic damage [42–44]. Proinflammatory cytokines are known to stimulate increased ROS production through the NADPH oxidase complex in RPE cells [45]. The hub genes also include genes from the chemokine pathway and the complement pathway, all of which indicate the immunologically charged environment of the RPE. PPI network of genes downregulated in aged RPE mainly involved histones, genes involved in phototransduction, and neural networks. Downregulation of histones in the aged RPE was recently reported by our group and further validated here as a critical age-related feature in the STRING network [30].

*In vitro* cultures have been utilized as aging research models for various cell types, yet their application to RPE models remains largely unexplored [46, 47]. Utilizing *in vitro* models offers distinct advantages such as lower cost, reduced animal use, and improved efficiency in screening for signaling pathways and therapeutic targets. One of our study objectives was to assess long-term hRPE cultures as a model for studying aging by comparing cellular characteristics and transcriptomic profiling with *in vivo* models. Previous studies have identified a TER of approximately 200 Ω.cm² as a feature of fully differentiated RPE culture, closely reflecting the physiology of native RPE [48–52]. *In vitro* RPE cultures typically achieve full differentiation by day 21, with well-established cell-cell junctions. Although TER continues to increase beyond day 21, the rate of this increase tends to slow [53, 54]. The current findings demonstrate that our long-term hRPE cultures maintain morphology, pigmentation, and polarity (TER of 731.2 (±6.6) Ω.cm² by day 28) even after extended culturing.

A notable gap in this field is the lack of studies investigating the global gene expression patterns of fetal hRPE to delineate the transition from maturation to aging; an important consideration when screening RPE cells for translational cell therapies. The monolayer culture system used in this study lacks interactions with the photoreceptor layer and the underlying Bruch’s membrane, factors which could influence the growth and maturation trajectory of hRPE cells [55]. However, a comparison of age-related gene expression changes between long-term *in vitro* models and physiologically aged mouse RPE revealed significant overlap. These changes include upregulation of genes involved in pro-inflammatory responses, matrix remodeling, and oxidative stress, alongside downregulation of genes related to visual processes. We demonstrate that the transcriptional programs underlying hRPE aging *in vitro* recapitulate gene expression patterns of *in vivo* models. Thus, long-term hRPE cultures provide a valuable tool for evaluating the effects of chronological aging on cellular functions and high-throughput screening of potential therapeutic targets. However, this study has limitations, including sample size (*n* = 4 per group) and the need for a more comprehensive transcriptome analysis to enhance the relevance of long-term *in vitro* models for aging research applications. To address this, our future work will focus on temporal transcriptome profiling at 1, 2, 4, and 6 months to gain a deeper understanding of the gene expression changes occurring with prolonged culturing of hRPE cells. While additional *in vitro* studies of mouse RPE cells would have been beneficial for comparison, technical challenges associated with long-term culturing of mouse RPE cells, as noted in previous studies, remain a significant barrier [56, 57].

Age-related alterations within RPE cells manifest as inflammation, immune activation, heightened oxidative stress, and diminished visual perception. Butler et al. [58] observed an upregulation of visual cycle genes in aging human donor RPE. However, our findings, align with previous microarray investigations in young and aged mouse RPE, delving into age-related alterations in gene expression [59, 60], and offers a more comprehensive analysis of the entire RPE/choroid transcriptome in young and aged mice. *In vitro* studies have shown that inflammatory cytokine treatment can downregulate genes critical for RPE function, including those involved in the visual cycle such as *CDH1*, *RPE65, RDH5, RDH10, TYR*, and *MERTK* [61]. This suggests that aging RPE, with elevated proinflammatory gene expression, experiences a decrease in visual cycle pathway gene expression in mouse RPE. These observations highlight species-specific transcriptional differences underlying age-related changes between humans and other model systems [26].

Leveraging RNA sequencing and bioinformatics tools provides a robust approach to uncover the molecular mechanisms underlying RPE aging. Data mining employing GO and KEGG pathway analysis tools reveals enriched genes linked to RPE aging. Through PPI visualization and analysis of hub genes using STRING and Cytoscape, we identified aging upregulated pathways and key nodes with significant influence on the network. In conclusion, our study delineates the gene signatures associated with proinflammatory responses, immune activation, and oxidative stress in RPE aging, laying the groundwork for therapeutic interventions aimed at delaying aging processes and targeting age-related retinal diseases.

## MATERIALS AND METHODS

### Animals

All mouse experiments complied with the guidelines established by the Association for Research in Vision and Ophthalmology for the Use of Animals in Ophthalmic and Vision Research. Male and female wild-type C57BL/6J mice aged between 2 and 3 months were included in the young group (*n* = 4), while mice aged between 20 and 24 months were assigned to the aged group (*n* = 4). The mice were purchased from the Jackson Laboratory and housed under standard conditions (23 ± 1°C, 40–50% humidity, and ad libitum access to food and water). The Institutional Animal Care and Use Committee of the East Tennessee State University approved the research protocol.

### Mouse RPE/choroid collection

RPE/choroid from young and aged mice were collected as described by Dubey et al. [30]. To harvest mouse RPE, isolated eyes were placed on a cold petri dish beneath a surgical microscope. The anterior sections of the eye were precisely excised and symmetrical radial cuts were made in a four-leaf pattern to carefully remove the neural retina from the mouse eyecup. The pigmented RPE/choroid layer was gently scraped from the sclera and was promptly transferred to TRIzol (Invitrogen 12183016, Carlsbad, CA, USA) for RNA isolation. The tissues were swiftly snap-frozen and preserved in liquid nitrogen if not used immediately.

### RNA isolation

For RNA sequencing analysis, RPE/choroid tissue (2 eyes/sample) was collected from young and aged mice (*n* = 4; two males and two females per group). RNA extraction was performed from TRIzol according to the manufacturer’s instructions. Following this, RNA purification and on-column genomic DNA digestion were carried out using the Pure Link RNA Micro Kit (Invitrogen 12183016, Carlsbad, CA, USA). The quality of RNA samples was evaluated using the Agilent 2100 Bioanalyzer system (Agilent, Santa Clara, CA, USA), ensuring a median concentration of 40 ng/µl and RIN values >9 to facilitate library preparation.

### Library preparation and sequencing

Library construction and sequencing on the Illumina NovaSeq X Plus platform were carried out at the Rush University Core Genomics Facility and the Roy J. Carver Biotechnology Center, University of Illinois, at Urbana-Champaign, respectively. The samples underwent a DNAse treatment and clean-up using the Qiagen RNase-Free DNase Set (Catalog: 79254) paired with the Qiagen RNeasy^®^ Mini QIAcube Kit (Catalog: 74116). A normalized total of about 200 ng went into the treatment/cleanup and was carried out per the manufacturer’s instructions on the QIAcube Connect instrument. The samples were assessed for quality post-treatment using High Sensitivity RNA ScreenTape Analysis by Agilent (catalog: 5067-5579 and 5067-5580) and the samples were normalized to 30 ng for library preparation. Samples underwent rRNA depletion as part of the library preparation using Revvity NEXTFLEX^®^ RiboNaut rRNA Depletion Kit (catalog: NOVA-512963). Post depletion the Revvity NEXTFLEX^®^ Rapid Directional RNA-Seq Automation Kit 2.0 (catalog: NOVA-5198-53) was used to finish the library preparation. Both processes were carried out on the Revvity Sciclone^®^ G3 NGSx iQ^™^ Workstation. Preliminary sequencing data was generated using the Illumina Miniseq^™^ and used for normalization of libraries prior to final sequencing via Illumina’s NovaSeq^™^ X Plus Sequencing System. To prevent index switching, Unique Dual Indexes (UDIs) were used for barcoding the libraries. The library pool was further quantitated by qPCR on a BioRad CFX Connect Real-Time System (BioRad Laboratories, Inc., Hercules, CA, USA). The pooled barcoded libraries were multiplexed and loaded on one 10B lane on a NovaSeq X Plus for cluster formation and sequencing. The libraries were sequenced from both ends of the fragments for a total of 150 bp from each end. The fastq read files were generated and demultiplexed with the bcl2fastq v2.20 Conversion Software (Illumina, San Diego, CA, USA).

### RNA-seq data mapping and analysis

Paired-end 150 bp raw reads were trimmed to eliminate Truseq adapters and bases from the 3’ end with quality scores less than 20 using cutadapt (ver 4.4) [62]. Trimmed reads shorter than 40 bp were discarded. Subsequently, trimmed reads were aligned to the *Mus musculus* (house mouse) genome assembly GRCm39 (mm39) from Genome Reference Consortium (GCA_000001635.9 GCF_000001635.27) using STAR [63]. The expression level of ENSEMBL genes was quantified using FeatureCounts [64]. Differential expression statistics were computed using edgeR [65], using raw expression counts obtained from quantification. Normalized expression was computed as log2 CPM (counts per million), including a TMM normalization and batch effect correction. All *p*-values were adjusted for multiple testing using the false discovery rate (FDR) correction of Benjamini and Hochberg. Principal component analysis of normalized read counts of the whole transcriptome dataset was conducted to identify the primary source of variation, using the “prcomp” function in the R statistical environment (R v.4.3.1). A volcano plot of DEGs was generated using the EnhancedVolcano R package. Hierarchical clustering and heat maps were constructed with log2-transformed normalized read counts utilizing the heatmap.2 function in R.

### Gene ontology and kyoto encyclopedia of genes and genomes (KEGG) enrichment analyses of DEGs

GO and KEGG pathway analyses were carried out using the web tool ShinyGo (ShinyGO 0.80) [66]. To functionally classify the DEGs, we identified over-represented GO terms in three categories, namely biological process, molecular function, and cellular component categories. Enriched GO terms were visualized as bar charts, with a significance threshold of FDR <0.05. Additionally, KEGG [67] pathway enrichment analysis was conducted with an FDR cutoff of 0.05 for significance and the Pathview [68] visualization tool was used to map the RNA-seq data.

### STRING-PPI and hub gene analysis

The STRING (v12.0) [23] online tool was used for predicting the PPI of proteins encoded by the upregulated and downregulated DEGs. Subsequently, a PPI network was constructed using the STRING database, with a high confidence score of ≥0.9 as the cutoff criterion. The outcomes of the STRING analysis were further processed using the clusterMaker2 plugin of Cytoscape tool (v3.8.2) [69, 70]. The large PPI network was subclustered by employing the Markov clustering (MCL) algorithm to form biologically relevant tightly linked protein networks, with the default granularity parameter (inflation value) set to 4. Hub genes within the PPI network were identified using the maximal clique centrality (MCC) algorithm of the CytoHubba [24] plugin of Cytoscape tool. Wikipathways [71] were imported into Cytoscape using the Wikipathways app, and RNA-seq data were overlaid using the built-in IdMapper functionality.

### qPCR

Total RNA was extracted from hRPE cells and mice RPE/choroid tissues as described previously [72] and cDNA was synthesized using the High Capacity cDNA Reverse Transcription Kit (Applied Biosystems 4368814, Lithuania) according to the manufacturer’s instructions. The qPCR was performed as described previously [72] using the primer list in Supplementary Table 3. Briefly, 20 ng cDNA was amplified using the Power SYBR Green PCR Master Mix (Applied Biosystems 4368706, Lithuania) and StepOnePlus Real-Time PCR System (Applied Biosystems, Lithuania). Relative gene expression changes were calculated using either GAPDH or Actin as the reference gene. The fold change was calculated by determining the ratio of mRNA levels to control values using the Δ threshold cycle (Ct) method (2^−ΔΔCt^).

### Immunofluorescence

Frozen mouse eye sections were processed for immunofluorescence as described previously [30]. After initial fixing and permeabilization, sections were blocked for 1 hour in a solution containing 4% normal goat serum and 3% bovine serum albumin in 1X PBS. The sections were then incubated with primary antibodies overnight at 4ºC, followed by incubation with fluorescently tagged secondary antibodies for 1 h at RT. Nuclei were counterstained with Hoechst (1:10,000; Invitrogen) for 5 minutes. Primary antibodies used included anti-Histone H1.0 antibody (1:250; Cat# ab125027, Abcam), anti-C4 b/d antibody (1:250; Cat# NB200-541, Novusbio), and Isotype controls. Images were captured at 40X magnification using a TCS SP8 confocal microscope (Leica).

### Establishment of long-term hRPE cultures and trans-Epithelial electrical resistance (TER) measurement

For long-term cultures, primary fetal human RPE cells (*n* = 3 isolates; Lonza, H-RPE 00194987, Walkersville, MD, USA) were seeded on fibronectin (356008, Corning, Bedford, MA, USA) coated 12 mm-polyester (PET) transwell inserts with 0.4 mm pores in a 12-well plate (#3460, Costar, Kennebunk, ME, USA) with 1 × 10^5^ cells per well. These cells were initially cultured in RPE culture medium (RPECM, alpha-MEM plus 1% N1 Supplement, 1% Glutamine-Penicillin-Streptomycin, 1% non-essential amino acids, 250 mg/L taurine, 20 mg/L hydrocortisone, 0.013 mg/L triiodo-thyronine) containing 10% FBS and maintained in a humidified atmosphere at 37°C with 5% CO_2_ and 95% air. After two days, when cells attained confluency, the FBS in the RPE medium was reduced to 1%. TER of hRPE cells was measured weekly after seeding transwell inserts using the EVOM2 Epithelial Volt-Ohm Meter (World Precision Instruments, Sarasota, FL, USA). In each plate, one coated insert without cells was measured as blank, and the final resistance was calculated by multiplication of net resistance (Total – blank, Ω) with effective membrane area (cm^2^).

### ZO-1 immunostaining

Long-term human RPE cultures were fixed with 4% paraformaldehyde for 15 minutes and then permeabilized in PBS with 0.25% Triton X-100 for 15 min at room temperature. Cells were then blocked in PBS with 4% normal goat serum and 3% bovine serum albumin for 1 h followed by incubation with anti-ZO-1 primary antibody (1:200; Cat# 40-2200, Invitrogen, Rockford, IL, USA) in blocking buffer overnight at 4°C. After three serial PBS washes, cells were labeled with goat anti-rabbit secondary antibodies conjugated with Alexa Fluor 488 (1:1000; Cat# A11070, Invitrogen, Eugene, OR) for 1 h and then co-stained with Hoechst 33342 (1:10000; Cat# H3570, Invitrogen, Eugene, OR, USA) for 5 min. Fluorescent and bright-field images were acquired with confocal fluorescent microscopy (TCS SP8, Leica, Wetzlar, Germany).

### Statistical analysis

GraphPad Prism (GraphPad Software, La Jolla, CA, USA) was used for routine statistical analysis. The results are presented as mean ± standard error of the mean (SEM) or mean ± standard deviation (SD). The significance levels were depicted as follows: ^*^p < 0.05; ^**^p < 0.01; ^***^p < 0.001. Group comparisons were performed using a non-parametric unpaired *t*-test.

### Data availability

Raw RNA-seq data from this manuscript were deposited to the GEO database under accession code GSE263427.

## Supplementary Materials

Supplementary Figures

Supplementary Tables
