# Supplementary material for: Deciphering age-related transcriptomic changes in the mouse retinal pigment epithelium
Source: Aging (Albany NY). 2025 Mar 4;17(3):657–84. doi: 10.18632/aging.206219 (PMC11984418; doi:10.18632/aging.206219)
Supplement: Supplementary Figures [file aging-17-206219-s001.pdf]

# SUPPLEMENTARY FIGURES

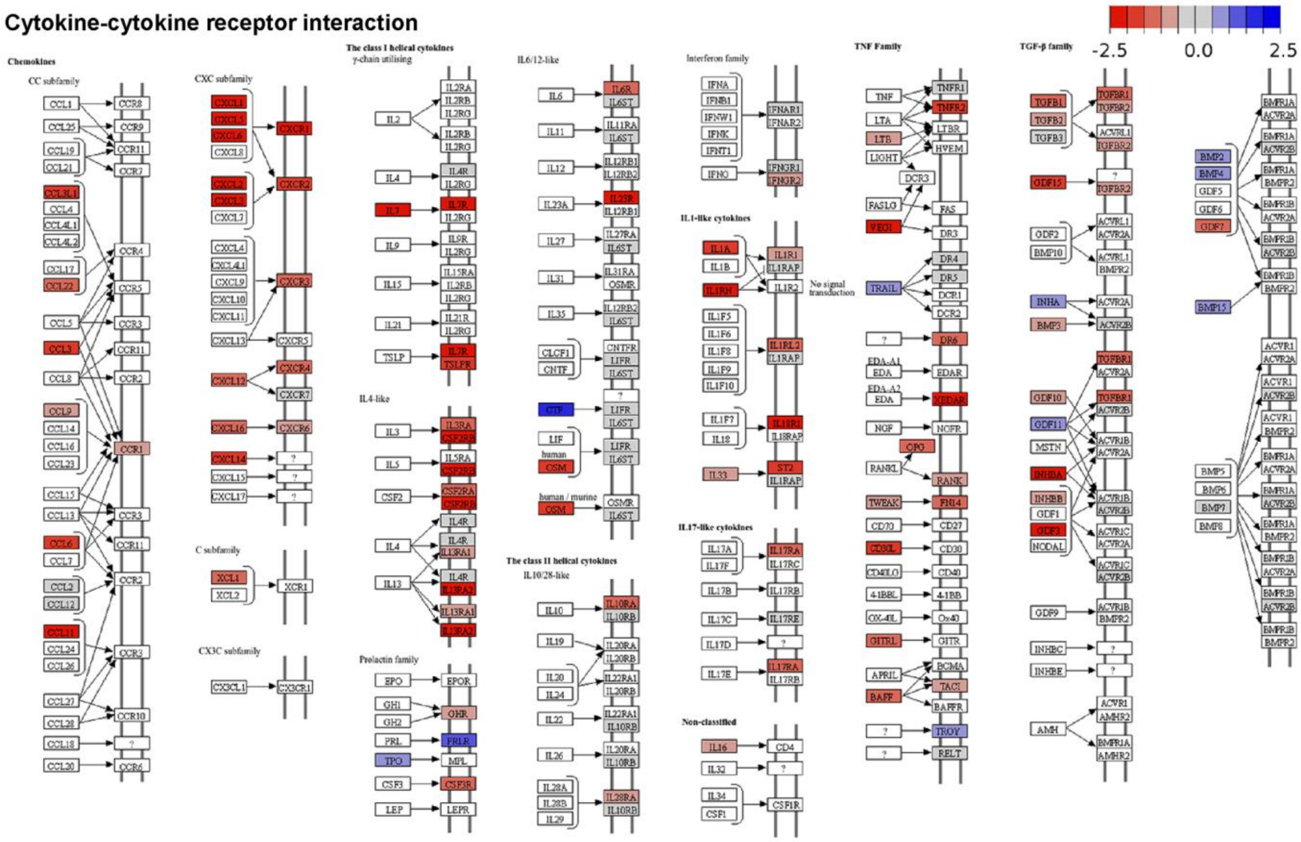


**Supplementary Figure 1. Pathway analysis.** Upregulation of several genes in the cytokine-cytokine receptor pathway in aged RPE.

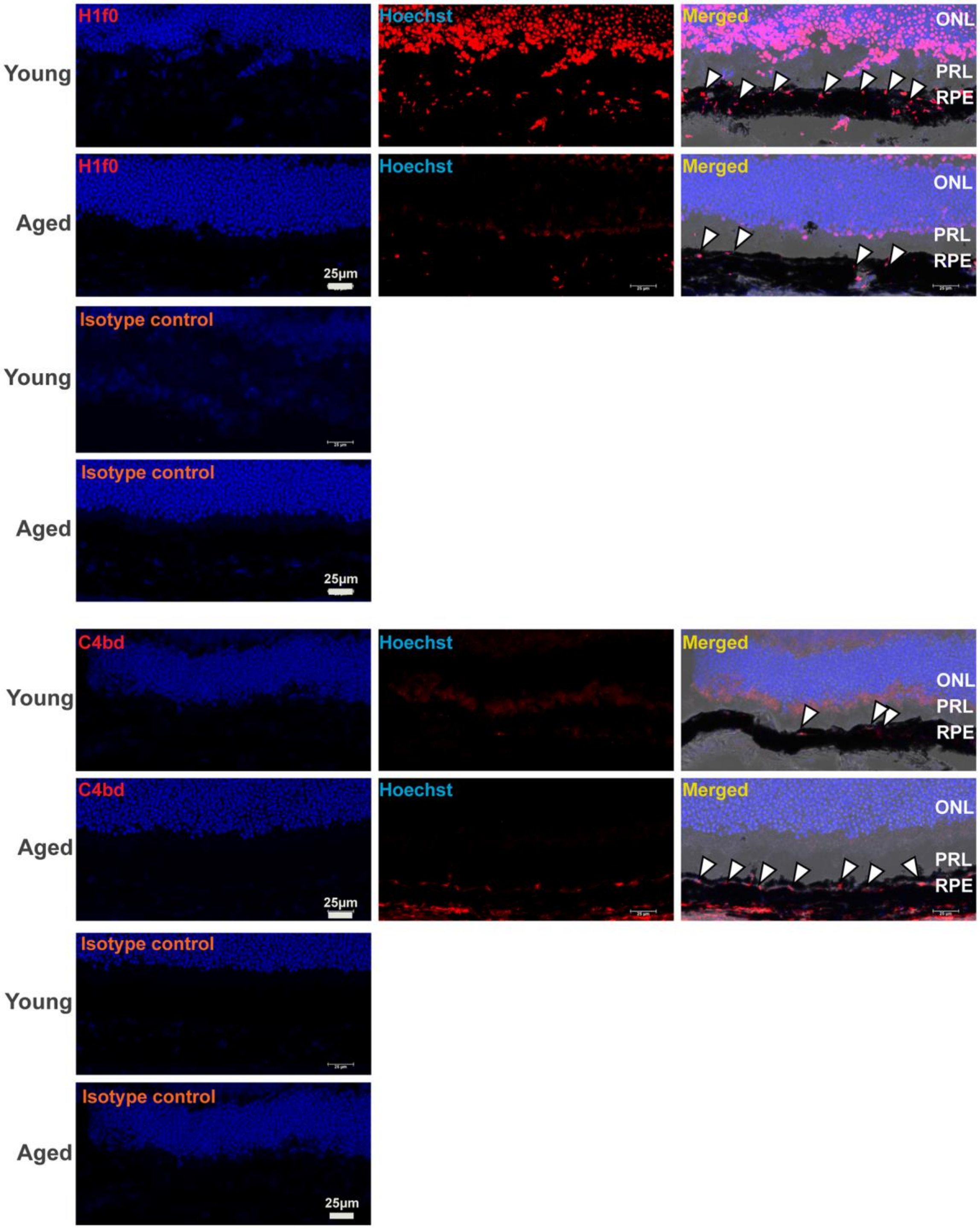


**Supplementary Figure 2. Representative fluorescence images of retinal cross-sections from young and aged mice illustrating the expression levels of H1f0 and C4b/d in the RPE layer.** Staining for the H1f0 histone variant (red) was stronger in the RPE of young mice (indicated by white arrows) compared to aged mice. Nuclei are stained with Hoechst (blue) in both young and aged retinas. Merged images combining the red and blue channels with brightfield illustrate the localization of H1f0 in the RPE layer. Conversely, the RPE of aged mice showed more intense staining for the complement factor C4b/d (red, white arrows) compared to young mice. The isotype control panel displayed no immunostaining and served as a negative control. Abbreviations: GCL: ganglion cell layer; IPL: inner plexiform layer; INL: inner nuclear layer; OPL: outer plexiform layer; ONL: outer nuclear layer; PRL: photoreceptor layer; RPE: retinal pigment epithelium. Scale bar: 25 µm.
