# Supplementary material for: Deciphering age-related transcriptomic changes in the mouse retinal pigment epithelium
Source: Aging (Albany NY). 2025 Mar 4;17(3):657–84. doi: 10.18632/aging.206219 (PMC11984418; doi:10.18632/aging.206219)
Supplement: Supplementary Tables [file aging-17-206219-s002.pdf]

SUPPLEMENTARY TABLES

Supplementary Table 1. List of top 100 PC1 loading genes and GO analysis of positive and negative loading genes.

PC1 loading genes		
**Sl. No**	**PC1_positive genes**	**PC1_negative genes**
1	Mfge8	Gm52669
2	Il7r	Adamtsl2
3	Mamdc2	Tgtp2
4	Serpina3n	4933429O19Rik
5	Anpep	Gabra4
6	Clec4d	Gm41620
7	Gpnmb	Alkal2
8	Cd68	Nxnl2
9	Cdo1	Wdr72
10	Atf3	Adgrl3
11	Gm14461	Gm46454
12	Fabp5	Opn4
13	Orm1	Gm41056
14	Stra6l	Nipal2
15	Lyz2	Rho
16	Trem2	Gm39041
17	Slc7a11	Gm34159
18	Pira2	Gm44867
19	Fabp4	Ccdc85a
20	Cd300lb	Gm40780
21	Cxcr1	Gm534
22	Atp6v0d2	A730049H05Rik
23	Lgals3	Gm46129
24	Csf2rb	Gm52634
25	Mcoln2	Krt8
26	Capg	Pcare
27	Itgam	Prph2
28	Tm4sf19	Gm52689
29	Cd200r4	Tcim
30	Gm20056	H4c17
31	Acp5	Rcvrn
32	Ms4a6d	Loxl4
33	Il1rn	Enpp2
34	Timp1	LOC118567855
35	Csf2rb2	Opn1mw
36	Tyrobp	Ush2a
37	Itgax	Lrit2
38	Cd84	Gpr139
39	Mmp12	4921534H16Rik
40	Slc11a1	St6galnac3
41	Dio2	Hs6st3
42	Pik3r5	Gbp4
43	Adam8	Tbc1d21

44	Havcr2	Atp7b
45	Adra1a	Gm15737
46	Galnt6	H19
47	Itgb2	Gm36746
48	Lyz1	Angptl4
49	Wfdc17	Rtl1
50	Pirb	Ano2
51	Hmcn2	Rab37
52	Gdf3	Gm46553
53	Bcl2a1b	Gm48957
54	Apod	Cacna1h
55	Galnt15	Gm52128
56	Ctss	Cldn10
57	Mpeg1	Gm15609
58	Kynu	Mme
59	Bpifb1	Rxfp3
60	Sfrp4	Mest
61	Laptm5	Sostdc1
62	4930430E12Rik	Chad
63	Cd53	Cutal
64	Pisd-ps3	Rian
65	Clec7a	Cdh6
66	Creg2	Trim12a
67	Mmp3	Gm51910
68	Cd36	Col1a1
69	Npy	Nr4a1
70	C3	Peg3
71	Pira1	Gdpd2
72	Tlr13	Tmprss2
73	H2-M2	Gm41253
74	Bcan	Clcnka
75	Olfr111	Tmem72
76	Acpp	3110053B16Rik
77	Necab2	Gja1
78	Ptx4	Dab1
79	Ly9	Gvin3
80	Cd5l	Gvin1
81	Ly75	Frem2
82	Lilrb4a	Gm33489
83	Prb1	Grik1
84	Igkc	LOC118568169
85	Lcn2	Gsg1l
86	Slc7a15	Adam7
87	Cybb	Gm33050
88	Slamf7	Gm42339
89	Lilr4b	Cd163
90	Glipr1	Gm40766
91	Jchain	Gm51909

92	Cma1	Matn4
93	Igha	Ifi213
94	Ighg2b	C430049B03Rik
95	Mmp13	Asic2
96	Tpsb2	Trhde
97	Mcpt4	Sox11
98	Gm2423	Ggt1
99	Serpine3	Dlk1
100	Erich5	Cilp2

PC1_positive loading genes GO

Category	Term ID	Term name	nGenes	Pathway genes	Enrichment FDR	Fold enrichment
GO:BP	GO:0006952	Defense response	37	1546	2.80E-16	5.8
GO:BP	GO:0006955	Immune response	33	1619	3.00E-12	5
GO:BP	GO:0002376	Immune system process	40	2544	4.30E-12	3.8
GO:BP	GO:0009607	Response to biotic stimulus	29	1439	1.70E-10	4.9
GO:BP	GO:0043207	Response to external biotic stimulus	28	1403	4.10E-10	4.9
GO:BP	GO:0051707	Response to other organism	28	1400	4.10E-10	4.9
GO:BP	GO:0098542	Defense response to other organism	24	1012	6.10E-10	5.8
GO:BP	GO:0006954	Inflammatory response	20	698	1.80E-09	7
GO:BP	GO:0044419	Biological process involved in interspecies interaction between organisms	28	1524	2.00E-09	4.5
GO:BP	GO:0006950	Response to stress	42	3637	7.40E-09	2.8

PC1_negative loading genes GO

Category	Term ID	Term name	nGenes	Pathway genes	Enrichment FDR	Fold enrichment
GO:BP	GO:0009583	Detection of light stimulus	7	52	3.30E-07	44.1
GO:BP	GO:0007601	Visual perception	9	155	6.60E-07	19
GO:BP	GO:0050953	Sensory perception of light stimulus	9	159	6.60E-07	18.6
GO:BP	GO:0007602	Phototransduction	5	30	1.20E-05	54.7
GO:BP	GO:0009581	Detection of external stimulus	7	128	3.20E-05	17.9
GO:BP	GO:0009582	Detection of abiotic stimulus	7	127	3.20E-05	18.1
GO:BP	GO:0009584	Detection of visible light	5	41	3.40E-05	40
GO:BP	GO:0018298	Protein-chromophore linkage	3	11	8.40E-04	89.4
GO:BP	GO:0009416	Response to light stimulus	8	318	9.40E-04	8.2
GO:BP	GO:0009628	Response to abiotic stimulus	14	1199	1.90E-03	3.8

Supplementary Table 2. GO analysis of upregulated and downregulated DE genes in aged mouse RPE/choroid.

Category	Term ID	Term name	nGenes	Pathway genes	Enrichment FDR	Fold enrichment
**Aging upregulated DE genes**						
GO:BP	GO:0006955	Immune response	200	1580	7.50E-63	3.9
GO:BP	GO:0002682	Reg. of immune system proc.	182	1430	6.10E-57	3.9
GO:BP	GO:0006954	Inflammatory response	123	739	3.00E-49	5.1
GO:BP	GO:0006952	Defense response	180	1642	1.00E-46	3.4
GO:BP	GO:0001775	Cell activation	140	1070	4.90E-44	4
GO:BP	GO:0002684	Positive reg. of immune system proc.	128	897	4.90E-44	4.4
GO:BP	GO:0050776	Reg. of immune response	118	812	4.00E-41	4.5
GO:BP	GO:0045321	Leukocyte activation	127	949	1.10E-40	4.1
GO:BP	GO:0002252	Immune effector proc.	98	629	2.80E-36	4.8
GO:BP	GO:0002443	Leukocyte mediated immunity	69	392	3.70E-28	5.4
GO:CC	GO:0005615	Extracellular space	162	1714	1.50E-33	2.9
GO:CC	GO:0009986	Cell surface	117	992	3.40E-32	3.6
GO:CC	GO:0098552	Side of membrane	84	676	4.00E-24	3.8
GO:CC	GO:0009897	External side of plasma membrane	67	466	1.20E-22	4.4
GO:CC	GO:0030312	External encapsulating structure	57	505	2.80E-14	3.5
GO:CC	GO:0031012	Extracellular matrix	57	503	2.80E-14	3.5
GO:CC	GO:0062023	Collagen-containing extracellular matrix	42	373	2.40E-10	3.5
GO:CC	GO:0045121	Membrane raft	43	408	1.00E-09	3.2
GO:CC	GO:0098857	Membrane microdomain	43	409	1.00E-09	3.2
GO:CC	GO:0001772	Immunological synapse	14	44	3.60E-09	9.7
GO:MF	GO:0005102	Signaling receptor binding	124	1561	1.00E-17	2.4
GO:MF	GO:0140375	Immune receptor activity	27	130	6.30E-12	6.4
GO:MF	GO:0004175	Endopeptidase activity	48	478	2.00E-09	3.1
GO:MF	GO:0008233	Peptidase activity	58	670	4.10E-09	2.7
GO:MF	GO:0004896	Cytokine receptor activity	19	95	4.40E-08	6.1
GO:MF	GO:0004252	Serine-type endopeptidase activity	26	202	4.70E-07	3.9
GO:MF	GO:0008236	Serine-type peptidase activity	26	217	1.90E-06	3.7
GO:MF	GO:0008009	Chemokine activity	12	45	1.90E-06	8.2
GO:MF	GO:0017171	Serine hydrolase activity	26	222	2.30E-06	3.6
GO:MF	GO:0019955	Cytokine binding	20	144	5.40E-06	4.3
**Aging downregulated DE genes**						
**Category**	**Term ID**	**Term name**	**nGenes**	**Pathway genes**	**Enrichment FDR**	**Fold enrichment**
GO:BP	GO:0007601	Visual perception	16	155	2.50E-11	14.9
GO:BP	GO:0050953	Sensory perception of light stimulus	16	159	2.50E-11	14.5
GO:BP	GO:0009583	Detection of light stimulus	10	52	1.90E-09	27.8
GO:BP	GO:0009584	Detection of visible light	8	41	2.20E-07	28.2
GO:BP	GO:0009581	Detection of external stimulus	11	128	6.20E-07	12.4
GO:BP	GO:0009582	Detection of abiotic stimulus	11	127	6.20E-07	12.5
GO:BP	GO:0007602	Phototransduction	6	30	1.90E-05	28.9
GO:BP	GO:0035458	Cellular response to interferon-beta	6	57	8.60E-04	15.2
GO:BP	GO:0035456	Response to interferon-beta	6	66	1.80E-03	13.1
GO:BP	GO:0009416	Response to light stimulus	11	318	3.50E-03	5
GO:CC	GO:0001750	Photoreceptor outer segment	8	88	1.80E-05	13.1
GO:CC	GO:0097730	Non-motile cilium	10	163	1.80E-05	8.9

GO:CC	GO:0097731	9+0 non-motile cilium	9	127	1.80E-05	10.2
GO:CC	GO:0097733	Photoreceptor cell cilium	9	114	1.80E-05	11.4
GO:CC	GO:0001917	Photoreceptor inner segment	6	73	6.70E-04	11.9
GO:CC	GO:0030312	External encapsulating structure	13	505	2.10E-03	3.7
GO:CC	GO:0031012	Extracellular matrix	13	503	2.10E-03	3.7
GO:CC	GO:0005929	Cilium	15	723	5.50E-03	3
GO:CC	GO:0005604	Basement membrane	5	115	4.00E-02	6.3
GO:CC	GO:0005615	Extracellular space	23	1714	4.40E-02	1.9
GO:MF	GO:0008020	G protein-coupled photoreceptor activity	3	10	3.20E-03	43.4
GO:MF	GO:0015267	Channel activity	13	483	3.20E-03	3.9
GO:MF	GO:0015318	Inorganic molecular entity transmembrane transporter activity	16	703	3.20E-03	3.3
GO:MF	GO:0022803	Passive transmembrane transporter activity	13	483	3.20E-03	3.9
GO:MF	GO:0022836	Gated channel activity	11	324	3.20E-03	4.9
GO:MF	GO:0005216	Ion channel activity	12	439	3.60E-03	4
GO:MF	GO:0015075	Ion transmembrane transporter activity	17	825	3.60E-03	3
GO:MF	GO:0009881	Photoreceptor activity	3	12	3.60E-03	36.1
GO:MF	GO:0005044	Scavenger receptor activity	4	46	1.20E-02	12.6
GO:MF	GO:0097110	Scaffold protein binding	5	82	1.20E-02	8.8

Supplementary Table 3. List of qPCR primers.

List of qPCR primers used in this study		
**Primer name**	**Forward**	**Reverse**
**Mouse**		
Best1	CATCAGCACCTCGGTCTACAAG	GACAAGTTGGCAAACCACACCC
Bco2	AACACGCGGTTTCATGTGGTGG	CCATCATCCTGGCAGCACAGAT
C1qb	CAACCAGGCACTCCAGGGATAA	CCAACTTTGCCTGGAGTCCCAG
C1s1	CAAAGCGATGGACAGTGGAGCA	CCAAACACACTATTCTCTGGCTC
C3	GGCCTTCTCTCTAACAGCCA	ATGCTGACCCTGAGGTCAAA
C4b	GGAGAGTGGAACCTGTAGACAG	CACTCGAACACGAGTTGGCTTG
Cxcr1	CCATTCCGTTCTGGTACAGTCTG	GTAGCAGACCAGCATAGTGAGC
Cxcr4	GACTGGCATAGTCGGCAATGGA	CAAAGAGGAGGTCAGCCACTGA
Cyba	GCTCATCTGTCTGCTGGAGTATC	CGGACGTAGTAATTCCTGGTGAG
Cybb	TGGCGATCTCAGCAAAAGGTGG	GTACTGTCCCACCTCCATCTTG
Gria3	GCAATGACAGCTCATCCTCCGA	GCGCTCATTTCCTTCCAGTTGC
Gnai1	CTCGGAAGAGGAGTGTAAGCAG	GCAAGCACGAAAAGTTGGCGAG
Gnat1	GCTTGTGGAAGGACTCGGGTAT	AACGCAACACGTCCTGCTCAGT
Gpc3	CTGTGCTGGAACGGACAAGAAC	GTCAATGATCTGGCTAACCACCG
Grk4	GATGGAGTCTCTGCTGTAAAGCA	CCTTTCACCACAGAGAACTGCC
Gucy2f	GGTGCTCCTTTCTGCATGATGG	GGCATACTCAGGAGAAACCACG
H3c11	TTCTAGTGTACTGAGATGGCTCGT	GTAGCGGTGAGGCTTCTTCA
Hdac3	GGCCAGAAGCACCCAATGAGTTCTA	ACAATCATCAGGCCGTGAGAGTTTG
Hdac8	CAGAGGAACCCGCCAACAG	CGCAGATGCTGACATACTCGG
H1f0	GATGAGCCCAAAAGGTCGGT	CTTCTTGACAGGGGTGGCTT
Napepld	AATCACAGCGGCGTTCCAGGTT	ACTCGTAAGCCAGCCTCTCTCA
Ncf1	GCTGACTACGAGAAGAGTTCGG	CCTCGCTTTGTCTTCATCTGGC
Ncf2	GCAGAAGAGCAGTTGGCATTGG	CTGCCTCTCATTTGGACGGAAC

Ncf4	CAAAGACCTGCTAGCGCTCATG	CCACATCCTCATCTGACAGCAG
Rbp3	CACCTCCCATTCTGCTGGACAA	ACACCACTGGTCAGGATGGCTA
Rdh12	GTGGTCATCACAGGTGCCAACA	GGAGTTCTTGGTATCTGCTCGG
Rdh5	CCATTGTGGAGCCTGGCTTCTT	ATGATGCGGCGCTGTACTCGAA
Rdh8	GGAGGACAAGTGGATGTGCTAG	CTTCATGCCTGGAAGCACAGCT
Rpe65	AAGGCTCCTCAGCCTGAAGTCA	GAGAACCTCAGGTTCCAGCCAT
Tet1	CCATTCTCACAAGGACATTCACA	GCAGGACGTGGAGTTGTTCA
	**Human**	
hCCL5	CCTGCTGCTTTGCCTACATTGC	ACACACTTGGCGGTTCTTTCGG
hC4B	AGATGCGGTGTCCAAGGTTCTG	GTTGCCAGGTATTTCCAAGGTCC
hCCL26	GGGAGTGACATATCCAAGACCTG	CAGACTTTCTTGCCTCTTTTGGTA
hCCL8	TATCCAGAGGCTGGAGAGCTAC	TGGAATCCCTGACCCATCTCTC
hCXCR4	CTCCTCTTTGTCATCACGCTTCC	GGATGAGGACACTGCTGTAGAG
hCYBA	ACCAGGAATTACTATGTTCGGGC	TAGGTAGATGCCGCTCGCAATG
hNCF2	ACTACTGCCTGACTCTGTGGTG	CCTCCACTTGGCTGCCTTTCTT
hBEST1	TGCCAACCTGTCAATGAAGGCG	TCCAGTCGTAGGCATACAGGTG
hCXCL8	GAGAGTGATTGAGAGTGGACCAC	CACAACCCTCTGCACCCAGTTT
hH4A,N,O	GAGACAACATTCAGGGCATCAC	GAGGCCAGAGATCCGCTTAA
hH4C	AAGTTAAGAGTTGTTGTTTGTCTTCG	CCACCTTTGCCTCTACCAGA
hH4D,E	TGGGTGAGACTCCTCTTGCT	AAGACCCTTCCCGCCTTT
hHIST1H1B	CTAAGGAGCGCAATGGCCTTT	CTTCGGAGTCTTCTTCACTGC
hHIST1H2AE	CTACTCCGAACGAGTCGGG	GATGGTCACGCGACCTAGAAG
hHIST1H2BF	ACCTGCTAAGTCCGCTCCT	CTACGCTTGCGCTTCTTACCA
hHIST1H3A	ACTGCTCGGAAGTCTACTGGT	GCGCTGGAAAGGTAGTTTACGA
hHIST1H3B	ATGGCTCGTACTAAACAGACAGC	TTCCGAATCAGCAACTCGGTC
hHIST1H3D	CCATTCCAGCGTCTAGTCCG	TCTGAAAACGCAGATCAGTCTTG
hHIST2H2AB	CCATCTGCAACTAGCCGTGAG	CAGGCTTGTGACTCTCCGT
hICAM1	AGCGGCTGACGTGTGCAGTAAT	TCTGAGACCTCTGGCTTCGTCA
hIL6	ATCTGGATTCAATGAGGAGACTTG	GGAACTGGATCAGGACTTTTGTACT
hMMP12	GATGCTGTCACTACCGTGGGAA	CAATGCCAGATGGCAAGGTTGG
hRPE65	TTTGGCACCTGTGCTTTCCCAG	GTTGGTCTCTGTGCAAGCGTAG
